# The Annual American Men’s Internet Survey of Behaviors of Men Who Have Sex With Men in the United States: 2015 Key Indicators Report

**DOI:** 10.2196/publichealth.7119

**Published:** 2017-03-25

**Authors:** Maria Zlotorzynska, Patrick Sullivan, Travis Sanchez

**Affiliations:** ^1^ Rollins School of Public Health Department of Epidemiology Emory University Atlanta, GA United States

**Keywords:** MSM, gay, homosexual, bisexual, HIV, STD, Internet, survey, surveillance, rapid surveillance report

## Abstract

The American Men’s Internet Survey (AMIS) is an annual Web-based behavioral survey of men who have sex with men (MSM) living in the United States. This Rapid Surveillance Report describes the third cycle of data collection (September 2015 through April 2016; AMIS-2015). The key indicators are the same as previously reported for AMIS (December 2013-May 2014, AMIS-2013; November 2014-April 2015, AMIS-2014). The AMIS survey methodology has not substantively changed since AMIS-2014. MSM were recruited from a variety of websites using banner advertisements and email blasts. Additionally, participants from AMIS-2014 who agreed to be recontacted for future research were emailed a link to the AMIS-2015 survey. Men were eligible to participate if they were age 15 years and older, resided in the United States, provided a valid US ZIP code, and reported ever having sex with a man. We examined demographic and recruitment characteristics using multivariable regression modeling (*P*<.05) stratified by participants’ self-reported human immunodeficiency virus (HIV) status. The AMIS-2015 round of data collection resulted in 10,217 completed surveys from MSM representing every US state and Puerto Rico. Participants were mainly non-Hispanic white, older than 40 years, living in the US South, living in urban areas, and recruited from general social networking websites. Self-reported HIV prevalence was 9.35% (955/10,217). Compared to HIV-negative/unknown status participants, HIV-positive participants were more likely to have had anal sex without a condom with any male partner in the past 12 months (75.50%, 721/955 vs 63.09%, 5843/9262, *P*<.001) and more likely to have had anal sex without a condom with a serodiscordant or unknown status partner (34.45%, 329/955 vs 17.07%, 1581/9262, *P*<.001). The reported use of marijuana and other illicit substances in the past 12 months was higher among HIV-positive participants than HIV-negative/unknown status participants (marijuana use: 24.61%, 235/955 vs 22.96%, 2127/9262; other illicit substance use: 28.59%, 273/955 vs 17.51%, 1622/9262, respectively; both *P*<.001). Most HIV-negative/unknown status participants (79.11%, 7327/9262) reported ever having a previous HIV test, and 55.69% (5158/9262) reported HIV testing in the past 12 months. HIV-positive participants were more likely to report sexually transmitted infection (STI) testing and diagnosis compared to HIV-negative/unknown status participants (STI testing: 71.73%, 685/955 vs 38.52%, 3568/9262; STI diagnosis: 25.65%, 245/955 vs 8.12%, 752/9262, respectively; both *P*<.001).

## Introduction

The American Men’s Internet Survey (AMIS) is an annual online behavioral survey of men who have sex with men (MSM) who live in the United States. The methods have been previously published [1,2]. This supplemental report updates that previous manuscript with the most current data available from AMIS (AMIS-2015). Methods in AMIS-2015 are unchanged from the previously published manuscript unless otherwise noted.

## Methods

### Recruitment and Enrollment

As in the prior year, AMIS participants were recruited through convenience sampling from a variety of websites using banner advertisements or email blasts to website members (hereafter referred to generically as “ads”). The survey was not incentivized. Data on the number of clicks on all banner ads were obtained directly from the websites. In AMIS-2014, data on the number of clicks on geospatial social networking banner ads were instead obtained by counting the number of clicks on the survey landing page. Men who clicked on the ads were taken directly to the survey website hosted on a secure server administered by SurveyGizmo (Boulder, CO, USA). Participants were also recruited by emailing participants from the previous cycle of AMIS (AMIS-2014) who consented to be recontacted for future studies. To be eligible for the survey, participants had to be 15 years of age or older, consider themselves to be male, and report that they had oral or anal sex with a man at least once in the past (hereafter referred to as MSM). Persons who reported being younger than 15 years of age or refused to provide their age were not asked any other screening questions. Those MSM who met the eligibility criteria and consented to participate in the study started the online survey immediately. The full questionnaire for AMIS-2015 is presented in Multimedia Appendix 1.

AMIS-2015 ran from September 2015 through April 2016, and resulted in 137,608 persons clicking on the ads and landing on the study’s recruitment page (Table 1). Most persons who clicked on the ads were from general social networking websites (66,500/137,608, 48.33%). Of the 1248 participants who completed the AMIS-2014 survey and were emailed links to the AMIS-2015 survey, 9.13% (114/1248) clicked on the link. One-third (33.58%, 46,207/137,608) of those who landed on the study’s page started the screening process and 56.09% (25,919/46,207) of those were eligible. The most common reason for ineligibility was not ever having male-male sex. More than three-quarters (78.52%, 20,351/25,919) of those who were eligible consented to participate in the survey. There were 2291 of 20,351 (11.26%) surveys determined to likely be from duplicate participants. Deduplication of survey responses was performed in the same manner as in previous AMIS cycles [1,2]. Among unduplicated surveys, almost two-thirds (64.21%, 11,597/18,060) were considered successful (ie, observations with no missing values for the first question of at least two consecutive sections). Most successful surveys were among men who reported having sex with another man in the past 12 months (89.07%, 10,330/11,597). Finally, 1.09% (113/10,330) of the sample was found to have provided an invalid ZIP code and was excluded from the final analytical sample.

**Table 1:** Recruitment outcomes for the American Men’s Internet Survey, United States, 2015.

Recruitment outcomes		Total	Recruitment type				
			Gay social networking (n=1)	General gay interest (n=2)	General social networking (n=4)	Geospatial social networking (n=2)	AMIS-2014 participants
Clicked ad, n		137,608	4680	3968	66,500	62,261	199
Screened,^a^ n (%)		46,207 (33.58)	3671 (78.44)	1165 (29.36)	30,581 (45.99)	10,630 (17.07)	160 (80.40)
Ineligible,^b^n (%)		20,288 (43.91)	740 (20.16)	463 (39.74)	16,206 (52.99)	2868 (26.98)	11 (6.88)
	Not age ≥15 years^c^	14,246 (70.22)	615 (83.11)	369 (79.70)	11,056 (68.22)	2197 (76.60)	9 (81.82)
	Not male^c^	15,255 (75.19)	559 (75.54)	381 (82.29)	11,800 (72.81)	2505 (87.34)	10 (90.91)
	Not ever MSM^c^	19,804 (97.61)	620 (83.78)	454 (98.06)	16,046 (99.01)	2673 (93.20)	11 (100.00)
	Nonresident^c^	15,502 (76.41)	624 (84.32)	377 (81.43)	11,469 (70.77)	2573 (89.71)	9 (81.82)
Eligible,^b^ n (%)		25,919 (56.09)	2931 (79.84)	702 (60.26)	14,375 (47.01)	7762 (73.02)	149 (93.13)
Consented,^d^ n (%)		20,351 (78.52)	2181 (74.41)	586 (83.48)	10,818 (75.26)	6623 (85.33)	143 (95.97)
Unduplicated,^e^ n (%)		18,060 (88.74)	2032 (93.17)	552 (94.20)	9410 (86.98)	5926 (89.48)	140 (97.90)
Success,^f^ n (%)		11,597 (64.21)	1568 (77.17)	426 (77.17)	6372 (67.72)	3104 (52.38)	127 (90.71)
MSM past 12 months,^g^ n (%)		10,330 (89.07)	1456 (92.86)	381 (89.44)	5425 (85.14)	2953 (95.14)	115 (90.55)
Valid ZIP code,^h^ n (%)		10,217 (98.91)	1451 (99.66)	381 (100.00)	5396 (99.47)	2875 (97.36)	114 (99.13)

^a^ Proportion is of total who clicked ad. Includes those who started the screening questionnaire.

^b^ Proportion is among total screened. Ineligible includes those who did not complete the screening questionnaire.

^c^ Proportion is among total ineligible. Includes those who may not have responded to the question. MSM: men who have sex with men.

^d^ Proportion is among eligible.

^e^ Proportion is among consented. Unduplicated removes participants who were marked as duplicates using IP address and demographic data matching.

^f^ Proportion is among unduplicated. Success removes participants who did not pass the test for survey completeness.

^g^ Proportion is among successes.

^h^ Proportion is among MSM in the past 12 months. Valid US ZIP codes were those that could be matched to the ZIP code-to-county crosswalk files created by the US Department of Housing and Urban Development. Any ZIP codes that could not be matched to this list were then hand-validated by checking against the ZIP code locator tool on the USPS website. ZIP codes that could not be found were classified as invalid.

Almost all these surveys (10,217/10,330, 98.91%) provided a valid US ZIP code. ZIP codes provided by participants were validated by merging them with the 2015 ZIP code-to-county crosswalk files created by the US Department of Housing and Urban Development [3]. Any ZIP codes that could not be matched to this list were then hand-validated by checking against the ZIP code locator tool on the United States Postal Service website [4]. ZIP codes that could not be found were classified as invalid. Overall, the completion rate was 7.4% (10,217/137,608), with an analytical sample consisting of 10,217 surveys out of 137,068 clicks.

### Measures and Analyses

For AMIS-2015 analyses, participants were categorized as either being AMIS-2014 participants who took the survey again or new participants from website/app types based on target audience and purpose: gay social networking (n=1), gay general interest (n=2), general social networking (n=4), and geospatial social networking (n=2). Recruitment outcomes and demographic characteristics for the AMIS-2014 participants are presented and for all behavioral outcomes, they are recategorized according to their original recruitment source. We do not provide the names of the websites/apps to preserve operator and client privacy, particularly where a category has only one operator. The participants who were eligible, consented, unduplicated, successful, reported male-male sex in the past 12 months, and provided a valid US ZIP code were included in analyses of participant characteristics and behavior.

For AMIS-2015, we created a more refined population density variable for each participant’s county of residence as determined by their ZIP code. The levels of the population density variable are from the National Center for Health Statistics (NCHS) Rural-Urban classification scheme [5]. The NCHS classifies counties into six categories: central (ie, inner city) or fringe (ie, suburban) areas of large metropolitan statistical areas (MSAs; population size ≥1,000,000), medium-sized MSAs (population size 250,000-999,999), small MSAs (population size <250,000), micropolitan area (counties that contain all or part of a city of 10,000 or more), and noncore (counties that do not contain any part of a city of 10,000 or more). We further collapsed these categories into a four-level urbanicity variable: urban (central), suburban (fringe), medium/small metropolitan, and rural (micropolitan and noncore).

The analysis methods for AMIS-2015 did not substantively differ from those previously published but are repeated in this report for clarity [1]. Overall, chi-square tests were used to identify whether participant characteristics significantly differed between recruitment sources. Multivariable logistic regression modeling was used to determine significant differences in behaviors based on self-reported human immunodeficiency virus (HIV) status while controlling for race/ethnicity, age group, National HIV Behavioral System (NHBS) city residency, and recruitment website type. MSAs included in NHBS in 2015 were Atlanta, GA; Baltimore, MD; Boston, MA; Chicago, IL; Dallas, TX; Denver, CO; Detroit, MI; Houston, TX; Los Angeles, CA; Miami, FL; Nassau-Suffolk, NY; New Orleans, LA, New York City, NY; Newark, NJ; Philadelphia, PA; San Diego, CA; San Francisco, CA; San Juan, PR; Seattle, WA; and Washington, DC. Self-reported HIV status was categorized as either HIV-positive or HIV-negative/unknown status, consistent with surveillance reports produced by NHBS [6]. HIV testing behaviors were only examined among those who did not report that they were HIV-positive and were also presented by participant characteristics. Multivariable logistic regression results are presented as Wald chi-square *P* values to denote an independently significant difference in the behavior for each subgroup compared to a referent group. Statistical significance was determined at *P*<.05.

## Results

**Figure 1:**
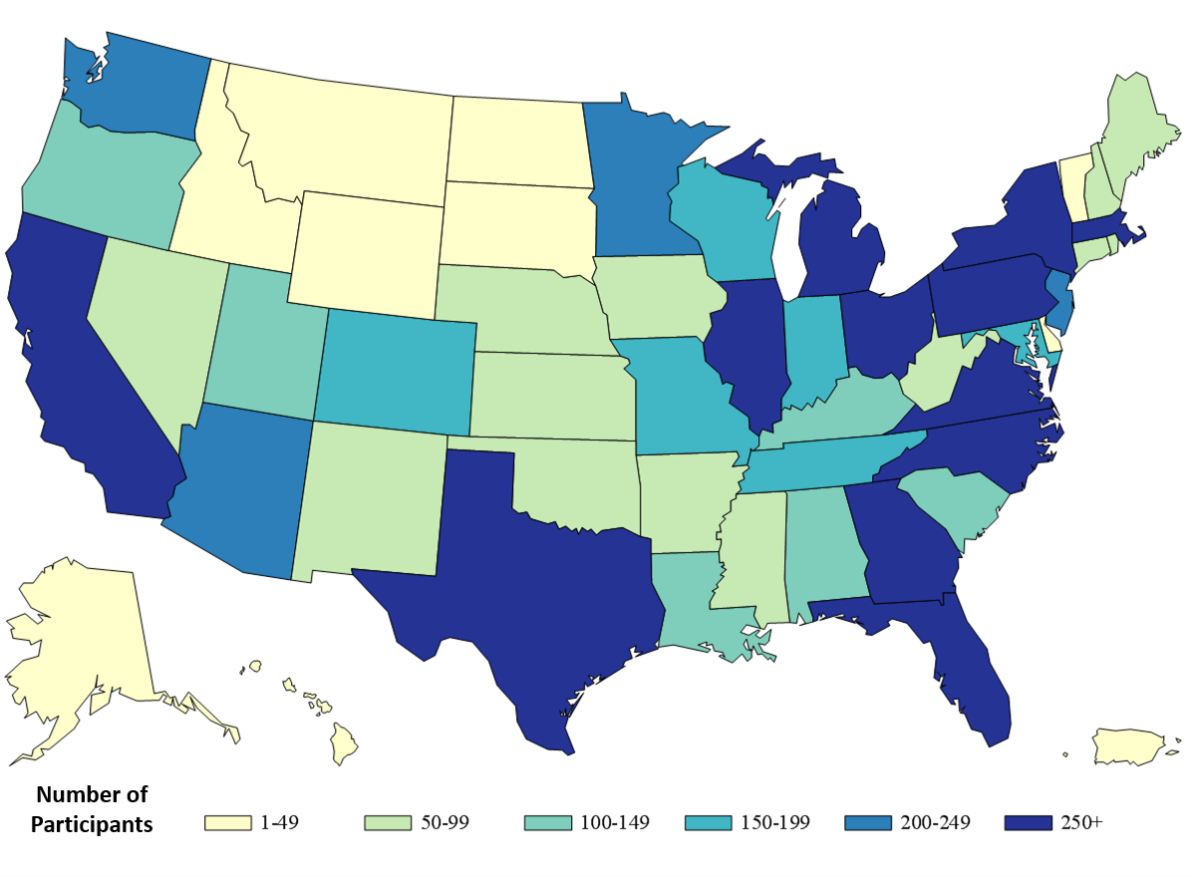
Number of MSM participants in the American Men’s Internet Survey by state, 2015.

Approximately seven in 10 (7291/10,217, 71.36%) participants included in this report were white and non-Hispanic, less than half were 40 years of age or older (4326/10,217, 42.34%), and their most common region of residence was the South followed by the West (Table 2). Participants were recruited from all US states and there were at least 100 participants from each of 28 states (Figure 1). Overall, 9.35% (955/10,217) of participants reported being HIV-positive, 69.91% (7143/10,217) reported being HIV-negative, and 20.74% (2119/10,217) reported having an unknown HIV status. All participant characteristics differed significantly based on where they were recruited (Table 2).

**Table 2:** Characteristics of MSM participants in the American Men’s Internet Survey by recruitment type, United States, 2015.

Participant characteristics		Total, n (%)	Recruitment type, n (%)					*P* ^a^
			Gay social networking (n=1)	General gay interest (n=4)	General social networking (n=4)	Geospatial social networking (n=2)	AMIS-2014 participants	
**Race/Ethnicity**								<.001
	Black, non-Hispanic	675 (6.61)	33 (2.27)	15 (3.94)	444 (8.23)	176 (6.12)	7 (6.14)	
	Hispanic	1387 (13.58)	73 (5.03)	36 (9.45)	755 (13.99)	511 (17.77)	12 (10.53)	
	White, non-Hispanic	7291 (71.36)	1271 (87.59)	301 (79.00)	3733 (69.18)	1899 (66.05)	87 (76.32)	
	Other or multiple races	864 (8.46)	74 (5.10)	29 (7.61)	464 (8.60)	289 (10.05)	8 (7.02)	
**Age (years)**								<.001
	15-24	2821 (27.61)	32 (2.21)	37 (9.71)	2155 (39.94)	581 (20.21)	16 (14.04)	
	25-29	1583 (15.49)	36 (2.48)	61 (16.01)	983 (18.22)	491 (17.08)	12 (10.53)	
	30-39	1487 (14.55)	112 (7.72)	86 (22.57)	516 (9.56)	740 (25.74)	33 (28.95)	
	≥40	4326 (42.34)	1271 (87.59)	197 (51.71)	1742 (32.28)	1063 (36.97)	53 (46.49)	
**Region**								.002
	Northeast	2038 (19.95)	304 (20.95)	72 (18.90)	1074 (19.90)	566 (19.69)	22 (19.30)	
	Midwest	2127 (20.82)	344 (23.71)	73 (19.16)	1152 (21.35)	530 (18.43)	28 (24.56)	
	South	3739 (36.60)	467 (32.18)	132 (34.65)	2000 (37.06)	1098 (38.19)	42 (36.84)	
	West	2305 (22.56)	335 (23.09)	103 (27.03)	1166 (21.61)	679 (23.62)	22 (19.30)	
US dependent areas		8 (0.08)	1 (0.07)	1 (0.26)	4 (0.07)	2 (0.07)	0 (0.0)	
**NHBS city resident^b^**								<.001
	Yes	3731 (36.52)	565 (38.94)	177 (46.46)	1855 (34.38)	1090 (37.91)	44 (38.60)	
	No	6486 (63.48)	886 (61.06)	204 (53.54)	3541 (65.62)	1785 (62.09)	70 (61.40)	
**Population density^c^**								<.001
	Urban	4101 (40.18)	572 (39.45)	189 (49.74)	2073 (38.45)	1214 (42.28)	53 (46.49)	
	Suburban	2041 (20.00)	363 (25.03)	71 (18.68)	1092 (20.26)	494 (17.21)	21 (18.42)	
	Small/ medium metropolitan	3076 (30.14)	387 (26.69)	97 (25.53)	1679 (31.14)	883 (30.76)	30 (26.32)	
	Rural	988 (9.68)	128 (8.83)	23 (6.05)	547 (10.15)	280 (9.75)	10 (8.77)	
**Self-reported HIV Status**								<.001
	Positive	955 (9.35)	108 (7.44)	26 (6.82)	411 (7.62)	395 (13.74)	15 (13.16)	
	Negative	7143 (69.91)	1102 (75.95)	302 (79.27)	3566 (66.05)	2080 (72.35)	93 (81.58)	
	Unknown	2119 (20.74)	241 (16.61)	53 (13.91)	1419 (26.32)	400 (13.91)	6 (5.26)	
Total		10,217	1451	381	5396	2875	114	

^a^ Chi-square test for difference in characteristics between recruitment types.

^b^ NHBS: National HIV Behavioral Surveillance System.

^C^ There were 11 participants living in US territories or provided military addresses, which could not have an NCHS urban/rural category assigned.

Most participants reported having anal sex without a condom with another man within the past 12 months (Table 3). Compared to HIV-negative/unknown status participants, those who were HIV-positive were significantly more likely to report anal intercourse without a condom (adjusted OR [AOR] 1.86, 95% CI 1.59-2.18), including with male partners who were of discordant or unknown status (AOR 2.75, 95% CI 2.36-3.20). Within each serostatus group, anal intercourse without a condom differed significantly by age group (HIV-positive and HIV-negative/unknown status participants), and recruitment website (HIV-negative/unknown status participants only). Anal intercourse without a condom with partners of discordant or unknown HIV status differed significantly by race/ethnicity (HIV-positive participants only), recruitment website (HIV-positive participants only), and age (HIV-negative/unknown status participants only).

**Table 3:** Sexual Behaviors with Male Partners of MSM Participants in the American Men’s Internet Survey, United States, 2015.

Participant characteristics			n	Sexual behaviors with male partners in the past 12 months			
				Anal intercourse without a condom		Anal intercourse without a condom with a partner of discordant or unknown HIV status	
				n (%)	*P* ^a^	n (%)	*P* ^a^
**HIV positive overall**			955	721 (75.50)	<.001^b^	329 (34.45)	<.001^b^
	**Race/Ethnicity**						
		Black, non-Hispanic	161	105 (65.22)	.08	35 (21.74)	.002
		Hispanic	152	113 (74.34)	.70	48 (31.58)	.92
		White, non-Hispanic	573	454 (79.23)	REF	221 (38.57)	REF
		Other or multiple races	69	49 (71.01)	.50	25 (36.23)	.37
	**Age (years)**						
		15-24	50	40 (80.00)	.83	18 (36.00)	.76
		25-29	107	92 (85.98)	.04	42 (39.25)	.37
		30-39	181	147 (81.22)	.91	68 (37.57)	.45
		≥40	617	442 (71.64)	REF	201 (32.58)	REF
	**NHBS city resident^c^**						
		Yes	422	325 (77.01)	.14	142 (33.65)	.83
		No	533	396 (74.30)	REF	187 (35.08)	REF
	**Recruitment type**						
		Gay social networking	108	79 (73.15)	.35	48 (44.44)	.18
		General gay interest	26	22 (84.62)	.30	12 (46.15)	.37
		General social networking	413	290 (70.22)	REF	137 (33.17)	REF
		Geospatial social networking	408	330 (80.88)	.59	132 (32.35)	.01
**HIV negative or unknown overall**			9262	5843 (63.09)	REF	1581 (17.07)	REF
	**Race/Ethnicity**						
		Black, non-Hispanic	514	316 (61.48)	.55	92 (17.90)	.44
		Hispanic	1235	804 (65.10)	.27	248 (20.08)	.05
		White, non-Hispanic	6718	4244 (63.17)	REF	1116 (16.61)	REF
		Other or multiple races	795	479 (60.25)	.05	125 (15.72)	.06
	**Age (years)**						
		15-24	2771	1713 (61.82)	<.001	524 (18.91)	.001
		25-29	1476	1072 (72.63)	<.001	257 (17.41)	.63
		30-39	1306	930 (71.21)	<.001	224 (17.15)	.37
		≥40	3709	2128 (57.37)	REF	576 (15.53)	REF
	**NHBS city resident^c^**						
		Yes	3309	2055 (62.10)	.17	570 (17.23)	.82
		No	5953	3788 (63.63)	REF	1011 (16.98)	REF
	**Recruitment type**						
		Gay social networking	1343	706 (52.57)	<.001	222 (16.53)	.49
		General gay interest	363	234 (64.46)	.59	60 (16.53)	.87
		General social networking	5028	3120 (62.05)	REF	816 (16.23)	REF
		Geospatial social networking	2528	1783 (70.53)	<.001	483 (19.11)	.06

^a^ Wald chi-square from multivariate logistic regression comparing behavior (yes vs no) among group with some characteristic compared to a referent (REF) group.

^b^ Wald chi-square from multivariate logistic regression comparing behavior (yes vs no) among HIV-positive participants compared to HIV-negative or unknown serostatus participants. Model controlled for race/ethnicity, age, NHBS residency, and recruitment type.

^C^ NHBS: National HIV Behavioral Surveillance System.

Almost one-quarter (235/955, 24.6%) of HIV-positive participants reported using marijuana in the past 12 months (Table 4). Compared to HIV-negative/unknown status participants, HIV-positive participants were significantly more likely to report use of marijuana (AOR 1.43, 95% CI 1.22-1.69) and other illicit substances in the past 12 months (AOR 2.20, 95% CI 1.88-2.59). Within each serostatus group, use of marijuana and other illicit substances differed significantly by age (HIV-positive and HIV-negative/unknown status participants), residence in an NHBS city (HIV-negative/unknown status participants only), and recruitment website type (HIV-negative/unknown status participants only). Marijuana use also differed significantly by recruitment website among HIV-positive participants. Use of other illicit substances differed significantly by race/ethnicity among HIV-negative/unknown status participants.

**Table 4:** Substance using behaviors of MSM participants in the American Men’s Internet Survey, United States, 2015.

Participant characteristics			n	Substance use behaviors in the past 12 months			
				Used marijuana		Used other substance(s)	
				n (%)	*P* ^a^	n (%)	*P* ^a^
**HIV positive overall**			955	235 (24.61)	<.001^b^	273 (28.59)	<.001^b^
	**Race/Ethnicity**						
		Black, non-Hispanic	161	40 (24.84)	.35	31 (19.25)	.06
		Hispanic	152	39 (25.66)	.89	49 (32.24)	.39
		White, non-Hispanic	573	144 (25.13)	REF	175 (30.54)	REF
		Other or multiple races	69	12 (17.39)	.07	18 (26.09)	.60
	**Age (years)**						
		15-24	52	15 (30.00)	.88	14 (28.00)	.48
		25-29	109	43 (40.19)	.003	43 (40.19)	.02
		30-39	187	53 (29.28)	.67	69 (38.12)	.19
		≥40	627	124 (20.10)	REF	147 (23.82)	REF
	**NHBS city resident^c^**						
		Yes	422	110 (26.07)	.35	125 (29.62)	.45
		No	533	125 (23.45)	REF	148 (27.77)	REF
	**Recruitment type**						
		Gay social networking	108	20 (18.52)	.89	28 (25.93)	.97
		General gay interest	26	4 (15.38)	.48	7 (26.92)	.93
		General social networking	413	90 (21.79)	REF	95 (23.00)	REF
		Geospatial social networking	408	121 (29.66)	.04	143 (35.05)	.14
**HIV negative or unknown overall**			9262	2127 (22.96)	REF	1622 (17.51)	REF
	**Race/Ethnicity**						
		Black, non-Hispanic	514	91 (17.70)	.06	67 (13.04)	.02
		Hispanic	1235	296 (23.97)	.78	220 (17.81)	.84
		White, non-Hispanic	6718	1570 (23.37)	REF	1200 (17.86)	REF
		Other or multiple races	795	170 (21.38)	.32	135 (16.98)	.94
	**Age (years)**						
		15-24	2771	854 (30.82)	<.001	543 (19.60)	.02
		25-29	1476	437 (29.61)	<.001	356 (24.12)	<.001
		30-39	1306	297 (22.74)	.16	254 (19.45)	.96
		≥40	3709	539 (14.53)	REF	469 (12.64)	REF
	**NHBS city resident^c^**						
		Yes	3309	793 (23.96)	.002	633 (19.13)	<.001
		No	5953	1334 (22.41)	REF	989 (16.61)	REF
	**Recruitment type**						
		Gay social networking	1343	187 (13.92)	.02	167 (12.43)	.13
		General gay interest	363	74 (20.39)	.92	57 (15.70)	.44
		General social networking	5028	1244 (24.74)	REF	859 (17.08)	REF
		Geospatial social networking	2528	622 (24.60)	.004	539 (21.32)	<.001

^a^ Wald chi-square from multivariable logistic regression comparing behavior (yes vs no) among group with some characteristic compared to a referent (REF) group.

^b^ Wald chi-square from multivariable logistic regression comparing behavior (yes vs no) among HIV-positive participants compared to HIV-negative or unknown serostatus participants. Model controlled for race/ethnicity, age, NHBS residency, and website type.

^c^ NHBS: National HIV Behavioral Surveillance System.

HIV testing behaviors were examined among those who did not report being HIV-positive (Table 5). Most participants (7327/9262, 79.11%) reported having been previously tested for HIV infection, and just over half (5158/9262, 55.69%) reported being tested in the past 12 months. HIV testing behavior, both ever tested and tested in past 12 months, differed significantly by age, residence in an NHBS city, and recruitment website type.

**Table 5:** HIV testing behaviors of HIV-negative or unknown status MSM participants in the American Men’s Internet Survey, United States, 2015.

Participant characteristics		n	HIV testing behaviors			
			HIV tested ever		HIV tested past 12 months	
			n (%)	*P* ^a^	n (%)	*P* ^a^
**Race/Ethnicity**						
	Black, non-Hispanic	514	445 (86.58)	.06	333 (64.79)	.02
	Hispanic	1235	948 (76.76)	.35	715 (57.89)	.37
	White, non-Hispanic	6718	5314 (79.10)	REF	3645 (54.26)	REF
	Other or multiple races	795	620 (77.99)	.99	465 (58.49)	.81
**Age (years)**						
	15-24	2771	1599 (57.70)	<.001	1286 (46.41)	<.001
	25-29	1476	1269 (85.98)	<.001	903 (61.18)	<.001
	30-39	1306	1160 (88.82)	<.001	858 (65.70)	<.001
	40 or older	3709	3299 (88.95)	REF	2111 (56.92)	REF
**NHBS city resident^b^**						
	Yes	3309	2774 (83.83)	<.001	2075 (62.71)	<.001
	No	5953	4553 (76.48)	REF	3083 (51.79)	REF
**Recruitment type**						
	Gay social networking	1343	1122 (83.54)	<.001	713 (53.09)	.005
	General gay interest	363	311 (85.67)	.87	189 (52.07)	.002
	General social networking	5028	3694 (73.47)	REF	2512 (49.96)	REF
	Geospatial social networking	2528	2200 (87.03)	<.001	1744 (68.99)	<.001
**Total**		9262	7327 (79.11)		5158 (55.69)	

^a^ Wald chi-square from multivariable logistic regression comparing behavior (yes vs no) among group with some characteristic compared to a referent (REF) group.

^b^ NHBS: National HIV Behavioral Surveillance System.

Compared to HIV-negative/unknown status participants, HIV-positive participants were significantly more likely to report sexually transmitted infection (STI) testing (AOR 4.00, 95% CI 3.43-4.68) and STI diagnosis (AOR 3.83, 95% CI 3.20-4.59) in the past 12 months (Table 6). The most common STI diagnosis among HIV-positive participants was syphilis (144/955, 15.1%), whereas gonorrhea was the most common STI diagnosis among HIV-negative/unknown status participants (427/9262, 4.61%). Among HIV-negative/unknown status participants, STI testing differed significantly by race/ethnicity, age, and residence in an NHBS city. Among both HIV-positive and HIV-negative/unknown status participants, STI testing differed significantly by recruitment website type and STI diagnosis differed significantly by age, NHBS city residence, and recruitment website type.

**Table 6:** Sexually transmitted infection testing and diagnosis of MSM participants in the American Men’s Internet Survey, United States, 2015.

Participant characteristics			n	STI History in the Past 12 Months			
				Tested for any STI^a^		Diagnosed with any STI^a^	
				n (%)	*P* ^b^	n (%)	*P* ^b^
**HIV positive overall**			955	685 (71.73)	<.001^c^	245 (25.65)	<.001^c^
	**Race/Ethnicity**						
		Black, non-Hispanic	161	116 (72.05)	.39	48 (29.81)	.10
		Hispanic	152	109 (71.71)	.29	50 (32.89)	.75
		White, non-Hispanic	573	413 (72.08)	REF	130 (22.69)	REF
		Other or multiple races	69	47 (68.12)	.32	17 (24.64)	.25
	**Age (years)**						
		15-24	50	44 (88.00)	.09	23 (46.00)	.02
		25-29	107	89 (83.18)	.45	43 (40.19)	.09
		30-39	181	152 (83.98)	.56	66 (36.46)	.77
		≥40	617	400 (64.83)	REF	113 (18.31)	REF
	**NHBS city resident^d^**						
		Yes	422	313 (74.17)	.10	128 (30.33)	.007
		No	533	372 (69.79)	REF	117 (21.95)	REF
	**Recruitment website type**						
		Gay social networking	108	71 (65.74)	.81	21 (19.44)	.40
		General gay interest	26	15 (57.69)	.25	3 (11.54)	.26
		General social networking	413	276 (66.83)	REF	79 (19.13)	REF
		Geospatial social networking	408	323 (79.17)	.006	142 (34.80)	.003
**HIV negative or unknown overall**			9262	3568 (38.52)	REF	752 (8.12)	REF
	**Race/Ethnicity**						
		Black, non-Hispanic	514	241 (46.89)	.01	57 (11.09)	.07
		Hispanic	1235	543 (43.97)	.92	150 (12.15)	.04
		White, non-Hispanic	6718	2458 (36.59)	REF	481 (7.16)	REF
		Other or multiple races	795	326 (41.01)	.30	64 (8.05)	.03
	**Age (years)**						
		15-24	2771	997 (35.98)	<.001	229 (8.26)	.46
		25-29	1476	753 (51.02)	<.001	169 (11.45)	<.001
		30-39	1306	639 (48.93)	.004	154 (11.79)	.15
		≥40	3709	1179 (31.79)	REF	200 (5.39)	REF
	**NHBS city resident^c^**						
		Yes	3309	1493 (45.12)	<.001	350 (10.58)	<.001
		No	5953	2075 (34.86)	REF	402 (6.75)	REF
	**Recruitment website type**						
		Gay social networking	1343	365 (27.18)	<.001	59 (4.39)	.04
		General gay interest	363	143 (39.39)	.88	23 (6.34)	.27
		General social networking	5028	1746 (34.73)	REF	313 (6.23)	REF
		Geospatial social networking	2528	1314 (51.98)	<.001	357 (14.12)	<.001

^a^ Sexually transmitted infection (STI) includes chlamydia, gonorrhea, and syphilis.

^b^ Wald chi-square from multivariable logistic regression comparing behavior (yes vs no) among group with some characteristic compared to a referent (REF) group.

^c^ Wald chi-square from multivariable logistic regression comparing behavior (yes vs no) among HIV-positive participants compared to HIV-negative or unknown serostatus participants. Model controlled for race/ethnicity, age, NHBS residency, and website type.

^d^ NHBS: National HIV Behavioral Surveillance System.

## Appendix

Multimedia Appendix 1 AMIS 2015 Questionnaire.

Multimedia Appendix 2 Recruitment and enrollment outcomes flow chart.

## Abbreviations

AMIS: American Men’s Internet Survey
HIV: human immunodeficiency virus
MSA: Metropolitan Statistical Area
MSM: men who have sex with men
NCHS: National Center for Health Statistics
NHBS: National HIV Behavioral Surveillance System
STI: sexually transmitted infection
