# Supplementary material for: The Annual American Men’s Internet Survey of Behaviors of Men Who Have Sex With Men in the United States: 2015 Key Indicators Report
Source: JMIR Public Health Surveill. 2017 Mar 25;3(1):e13. doi: 10.2196/publichealth.7119 (PMC5390111; doi:10.2196/publichealth.7119)
Supplement: Multimedia Appendix 1 [file publichealth_v3i1e13_app1.pdf]

American Men’s Internet Survey – 201

Landing page....................2

Eligibility Screener....................3

Consent....................5

Demographics....................7

Sexual Behavior with Female Partners in the Past 12 Months....................12

Sexual Behavior with Male Partners in the Past 12 Months....................14

Sexual Behavior with Last Male Partner....................19

Substance Use: Injection Drug Use....................32

Substance Use: Non-Injection Drug Use....................33

Alcohol and Drugs at Last Sex....................35

HIV Testing....................37

PrEP and PEP....................44

Experiences of Stigma....................51

Adverse Childhood Events....................56

STIs....................62

Prevention Activities....................65

Landing page....................1

Eligibility Screener....................2

Consent....................4

Demographics....................6

Sexual Behavior with Female Partners in the Past 12 Months....................11

Sexual Behavior with Male Partners in the Past 12 Months....................13

Sexual Behavior with Last Male Partner....................18

Substance Use: Injection Drug Use....................31

Substance Use: Non-Injection Drug Use....................32

Alcohol and Drugs at Last Sex....................34

HIV Testing....................36

PrEP and PEP....................43

Experiences of Stigma....................50

Adverse Childhood Events....................56

STIs....................61

Prevention Activities....................64

Landing page

EMORY

ROLLINS SCHOOL OF PUBLIC HEALTH

Join over 10,000 men in the U.S. by taking our annual sexual health survey!

SEX is the question

The answer is YOU

"Sex is the Question" collects information to help researchers better understand patterns of behavior – both sexual and health promoting – among men and to help ensure prevention resources have the greatest impact for the community. Click or tap the arrow or "Next" button below to be a part of this national effort!

Eligibility Screener

How old are you?*

____

Since October 2014, did you already complete at least part of Sex is the Question?

( ) No

( ) Yes

( ) I prefer not to answer

( ) Don't know

Do you consider yourself to be Hispanic or Latino?*

( ) No

( ) Yes

( ) I prefer not to answer

( ) Don't know

Which racial group or groups do you consider yourself to be in? Check all that apply.*

[ ] American Indian or Alaska Native

[ ] Asian

[ ] Black or African American

[ ] Native Hawaiian or Other Pacific Islander

[ ] White

[ ] I prefer not to answer

[ ] Does not apply

[ ] Don't know

What ZIP code do you live in?*

____

Do you consider yourself to be male, female, or transgender?*

( ) Male

( ) Female

( ) Transgender

( ) Don't know

( ) Prefer not to answer

Have you <u>ever</u> had vaginal sex (penis in the vagina) or anal sex (penis in the butt) with a woman?*

( ) No

( ) Yes

( ) I prefer not to answer

( ) Don't know

Have you <u>ever</u> had oral sex (mouth on the penis) with a man?*

( ) No

( ) Yes

( ) I prefer not to answer

( ) Don't know

Have you <u>ever</u> had anal sex (penis in the butt) with a man?*

( ) No

( ) Yes

( ) I prefer not to answer

( ) Don't know

Do you consider yourself to be:

( ) Homosexual or Gay

( ) Heterosexual or Straight

( ) Bisexual

( ) I prefer not to answer

( ) Don't know

Consent

Thank you for your interest in our survey. Please note:

1. Your answers are confidential: we don't have any information about who you are beyond the questions you answer.

2. This survey includes some personal questions. You can choose to not answer any questions that make you feel uncomfortable.

3. If you agree to be in this study, you will first be screened to see if you qualify to be in the study. If you qualify, you will be given the opportunity to complete the survey.

If you have any questions or comments you may contact the research staff at sexisthequestion@emory.edu.

Please read the information below about the study, and indicate whether you consent to participate in the study. *

( ) I consent to participate in the survey.

( ) I do not consent to participate in the survey.

Assent

Thank you for your interest in our survey. Please note:

1. Your answers are confidential: we don't have any information about who you are beyond the questions you answer.

2. This survey includes some personal questions. You can choose to not answer any questions that make you feel uncomfortable.

3. If you agree to be in this study, you will first be screened to see if you qualify to be in the study. If you qualify, you will be given the opportunity to complete the survey.

If you have any questions or comments you may contact the research staff at sexisthequestion@emory.edu.

Please read the information below about the study, and indicate whether you agree to participate in the study. *

( ) I have read the information below. I agree to participate in this study.

( ) I do not agree to participate in the survey.

Demographics

What is the highest level of education you completed?

( ) Never attended school

( ) Less than high school

( ) Some high school

( ) High school diploma or GED

( ) Some college, Associate's Degree, or Technical Degree

( ) College, post graduate or professional school

( ) I prefer not to answer

( ) Don't know

What was your household income last year from all sources before taxes? That is, the total amount of money earned and shared by all people living in your household.

( ) $0 to $19,999 annually ($0 to $1667 monthly)

( ) $20,000 to $39,999 annually ($1668 to $3333 monthly)

( ) $40,000 to $74,999 annually ($3334 to $6250 monthly)

( ) $75,000 or more annually ($6251 or more monthly)

( ) I prefer not to answer

( ) Don't know

Including yourself, how many people depend on this income?

____

How many of your dependents are under the age of 18?

____

In the past month:
Did you stay in a motel or hotel because you had nowhere else to sleep?

( ) No

( ) Yes

( ) I prefer not to answer

( ) Don't know

In the past month:
Did you double up or stay overnight with friends, relatives, or someone you didn’t know well because you didn’t have a regular, adequate, and safe place to stay at night?

( ) No

( ) Yes

( ) I prefer not to answer

( ) Don't know

In the past month:
Did you stay in a shelter or other facility that provides short-term housing for people who do not have their own place to sleep?

( ) No

( ) Yes

( ) I prefer not to answer

( ) Don't know

In the past month:
Did you stay overnight in a car, park, public place, abandoned building, bus or train station, or airport because you didn’t have a regular, adequate, and safe place to sleep?

( ) No

( ) Yes

( ) I prefer not to answer

( ) Don't know

What kind of health insurance or health care coverage do you currently have?

Health insurance--health plans people get through employment or purchased directly as well as government programs (like Medicare and Medicaid) that provide medical care or help pay medical bills.

Choose all that apply:

[ ] A private health plan purchased through an employer

[ ] A private health plan purchased through an exchange (i.e. Obamacare)

[ ] Medicaid or Medicare

[ ] Some other Medical Assistance program

[ ] TRICARE (CHAMPUS)

[ ] Veterans Administration coverage

[ ] Some other health care plan

[ ] I don't currently have any health insurance

[ ] I prefer not to answer

[ ] Don't know

In the past 12 months, have you seen a doctor, nurse, or other health care provider about your own health?

( ) No

( ) Yes

( ) I prefer not to answer

( ) Don't know

At any of those times you were seen by a doctor or health care provider, were you offered an HIV test? An HIV test checks whether someone has the virus that causes AIDS.

( ) No

( ) Yes

( ) I prefer not to answer

( ) Don't know

Did your doctor or health care provider talk to you about sex (gay or straight) or sexual health?

( ) No

( ) Yes

( ) I prefer not to answer

( ) Don't know

Do you consider yourself to be:

( ) Heterosexual or "Straight"

( ) Homosexual or Gay

( ) Bisexual

( ) I prefer not to answer

( ) Don't know

Outness

Have you ever told anyone that you are attracted to or have sex with men?

( ) No

( ) Yes

( ) I prefer not to answer

( ) Don't know

Who of the following people have you told that you are attracted to or have sex with men?

	**No**	**Yes**	**Does not apply**
Gay, lesbian, or bisexual friends	( )	( )	( )
Friends who are not gay, lesbian, or bisexual	( )	( )	( )
Family members	( )	( )	( )
Health care provider	( )	( )	( )
Employer	( )	( )	( )
Fellow employees	( )	( )	( )

Marriage

Have you ever been legally married?

( ) Yes

( ) No

Are you currently legally married?

( ) Yes

( ) No

What is the gender of the partner to whom you are legally married?

( ) Male

( ) Female

( ) Transgender (Male-to-Female)

( ) Transgender (Female-to-Male)

Sexual Behavior with Female Partners in the Past 12 Months

The next questions are about having sex with women. For these questions, "having sex" means oral, vaginal, or anal sex. Oral sex means mouth on the vagina or penis; vaginal sex means penis in the vagina; and anal sex means penis in the anus (butt).

In the past 12 months [SURVEY DATE] of 2014), what types of sex have you had with a woman? (Check all that apply.)

[ ] Oral sex

[ ] Vaginal sex

[ ] Anal sex

[ ] Some other type of sex

[ ] I have not had any type of sex with a woman in the past 12 months

[ ] Prefer not to answer

[ ] Don't know

These next questions are about the last time you had oral, vaginal or anal sex with a woman.

Was the woman you had sex with that last time a main partner (someone you felt committed to above anyone else) or a casual partner (someone you didn't feel committed to or don’t know very well)?

( ) Main sex partner

( ) Casual sex partner

( ) I prefer not to answer

( ) Don't know

When you had sex that last time, did you have either vaginal or anal sex?

( ) No

( ) Yes

( ) I prefer not to answer

( ) Don't know

The last time you had sex with a woman, did you have either vaginal or anal sex without using a condom?

( ) No

( ) Yes

( ) I prefer not to answer

( ) Don't know

The last time you had sex with this partner, did you know her HIV status?

( ) No

( ) Yes

( ) I prefer not to answer

What was her HIV status?

( ) HIV-negative

( ) HIV-positive

( ) Indeterminate

( ) I prefer not to answer

Sexual Behavior with Male Partners in the Past 12 Months

The next questions are about having sex with men. For these questions, "having sex" means oral or anal sex. Oral sex means he put his mouth on your penis or you put your mouth on his penis. Anal sex means you put your penis in his anus (butt) or he put his penis in your anus (butt).

How old were you the first time you had <u>oral sex</u> (mouth on the penis) with a man?

____

How old were you the first time you had <u>anal sex</u> (penis in the butt) with a man?

____

Do you consider yourself to be a top, bottom, or versatile?

( ) Top

( ) Bottom

( ) Versatile

( ) I prefer not to answer

( ) Don't know

In the past 12 months, what types of sex have you had with other men?

[ ] Oral sex

[ ] Anal sex

[ ] Some other type of sex

[ ] I have not had any type of sex with a man in the past 12 months

[ ] I prefer not to answer

[ ] Don't know

Please specify other type of sex:

____

In the past 12 months, since [system("date"),format="F"] of 2014, with how many different men have you had oral or anal sex?

____

In the past 12 months, since [system("date"),format="F"] of 2014, with how many different men have you had anal sex?

____

In the past 12 months, since [system("date"),format="F"] of 2014, with how many different men have you had oral sex?

____

Of the [question("value"), id="233"] men you had oral or anal sex with in the past 12 months, how many of them did you have anal sex with?

____

In the past 12 months, did you have anal sex without using a condom?

( ) No

( ) Yes

( ) I prefer not to answer

( ) Don't know

In the past 12 months, with how many of these [question("value"), id="487"] male anal sex partners did you have anal sex <u>without</u> using a condom?

____

In the past 12 months, with how many of these [question("value"), id="387"] male anal sex partners did you have anal sex <u>without</u> using a condom?

____

Sexual Behavior: Male Sex Partners (1 Partner)

In the past 12 months, this male partner was a:

( ) Main partner (someone you felt committed to above anyone else)

( ) Casual partner (someone you didn't feel committed to or don't know very well)

( ) I prefer not to answer

( ) Don't know

Did you know his HIV status?

( ) No

( ) Yes

( ) I prefer not to answer

What was his HIV status?

( ) HIV-negative

( ) HIV-positive

( ) Indeterminate

( ) I prefer not to answer

Sexual Behavior: Male Sex Partners (>1)

In the past 12 months, the [question("value"), id="233"][question("value"), id="487"] [question("value"), id="488"] male partners you told us about were:

( ) Only main partners (you felt committed to above anyone else)

( ) Only casual partners (you didn't feel committed to or don't know very well)

( ) Both main and casual partners

( ) I prefer not to answer

( ) Don't know

In the past 12 months, did you have anal sex <u>without using a condom</u> with a man whose HIV status you did not know?

( ) No

( ) Yes

( ) I prefer not to answer

( ) Don't know

Was this with a main or casual partner?

( ) Main partner

( ) Casual partner

( ) Both main and casual partners

( ) I prefer not to answer

( ) Don't know

In the past 12 months, did you have anal sex <u>without using a condom</u> with a man who was HIV positive?

( ) No

( ) Yes

( ) I prefer not to answer

( ) Don't know

Was this with a main or casual partner?

( ) Main partner

( ) Casual partner

( ) Both main and casual partners

( ) I prefer not to answer

( ) Don't know

In the past 12 months, did you have anal sex without using a condom with a man who was HIV negative?

( ) No

( ) Yes

( ) I prefer not to answer

( ) Don't know

Was this with a main or casual partner?

( ) Main partner

( ) Casual partner

( ) Both main and casual partners

( ) I prefer not to answer

( ) Don't know

Sexual Behavior with Last Male Partner

What are the initials of or a nickname for your last male sex partner?

____

When was the last time you had either oral or anal sex with [INITIALS/NICKNAME]?

Month:

( ) January

( ) February

( ) March

( ) April

( ) May

( ) June

( ) July

( ) August

( ) September

( ) October

( ) November

( ) December

Year:: ____

Was [INITIALS/NICKNAME] a main partner (someone you felt committed to above anyone else) or a casual partner (someone you didn't feel committed to or don't know very well)?

( ) Main sex partner

( ) Casual sex partner

( ) I prefer not to answer

( ) Don't know

That last time you had sex with [INITIALS/NICKNAME], did you have receptive anal sex where he put his penis in your anus (you were the bottom)?

( ) No

( ) Yes

( ) I prefer not to answer

( ) Don't know

During that last time you had receptive anal sex, did [INITIALS/NICKNAME] use a condom?

( ) No

( ) Yes, but not the whole time

( ) Yes, the whole time

( ) I prefer not to answer

( ) Don't know

When you had sex that last time, did you have insertive anal sex where you put your penis in his anus (you were the top)?

( ) No

( ) Yes

( ) I prefer not to answer

( ) Don't know

During insertive anal sex that last time, did you use a condom?

( ) No

( ) Yes, but not the whole time

( ) Yes, the whole time

( ) I prefer not to answer

( ) Don't know

The last time you had sex with [INITIALS/NICKNAME], did you know his HIV status?

( ) No

( ) Yes

( ) I prefer not to answer

What was [INITIALS/NICKNAME]'s HIV status?

( ) HIV-negative

( ) HIV-positive

( ) Indeterminate

( ) I prefer not to answer

What was [INITIALS/NICKNAME]'s age?

( ) 19 years or younger
( ) 20 to 24 years
( ) 25 to 29 years
( ) 30 to 34 years
( ) 35 to 39 years
( ) 40 to 44 years
( ) 45 to 49 years
( ) 50 to 54 years
( ) 55 to 59 years
( ) 60 to 64 years
( ) 65 to 69 years
( ) 70 to 74 years
( ) 75 to 79 years
( ) 80 years or older
( ) Don't know
( ) Prefer not to answer

Was [INITIALS/NICKNAME] younger than you, older than you, or the same age as you?

[ ] Younger
[ ] Older
[ ] Same age
[ ] I prefer not to answer
[ ] Don't know

Which of the following best describes [INITIALS/NICKNAME]'s ethnic background?

[ ] American Indian or Alaska Native
[ ] Asian
[ ] Black or African American

[ ] Hispanic or Latino

[ ] Native Hawaiian or other Pacific Islander

[ ] White

[ ] I prefer not to answer

[ ] Don't know

As far as you know, has [INITIALS/NICKNAME] ever injected drugs like heroin, cocaine, or speed?

Would you say he:

( ) Definitely did not

( ) Probably did not

( ) Probably did

( ) Definitely did

( ) I prefer not to answer

( ) Don't know

As far as you know, has [INITIALS/NICKNAME] ever used crystal meth (tina, crank, ice)?

( ) Definitely did not

( ) Probably did not

( ) Probably did

( ) Definitely did

( ) I prefer not to answer

( ) Don't know

Did you have sex with [INITIALS/NICKNAME] one time ('one night stand'), or more than one time?

( ) One time

( ) More than one time

( ) I prefer not to answer

( ) Don't know

How long have you been having a sexual relationship with [INITIALS/NICKNAME]?

____

( ) Days

( ) Months

( ) Years

Do you expect to have sex with [INITIALS/NICKNAME] again?

( ) Yes

( ) No

( ) Don't know

( ) I prefer not to answer

As far as you know, during the time you were having a sexual relationship with [INITIALS/NICKNAME], did he have sex with other people?

Would you say he:

( ) Definitely did not

( ) Probably did not

( ) Probably did

( ) Definitely did

( ) I prefer not to answer

( ) Don't know

During the time you were having a sexual relationship with [INITIALS/NICKNAME], did you have sex with other people?

( ) No

( ) Yes

( ) I prefer not to answer

( ) Don't know

Where did you first meet [INITIALS/NICKNAME]?

( ) Work

( ) School

( ) House party

( ) Mobile phone app (such as a gay chat, dating or hookup app)

( ) Internet

( ) Bar/Club

( ) Circuit party or rave

( ) Public sex environment (such as a bathhouse, sex club, sex resort, cruising area, private sex party, or adult bookstore)

( ) Place or worship (such as a church, synagogue, mosque)

( ) Other: ____

( ) I prefer not to answer

( ) Don't know

Partnership agreements for main partners

You indicated earlier that one or more of your male sexual partners was a main partner, someone you felt committed to above anyone else. The next set of questions are about your relationship with this person. If you have or had more than one main partner in the past 12 months, the questions are about the main partner you **most recently** had sex with.

To make the questions easier to ask, we'd like you to enter in this partner's initials or a nickname that is not his real name. If you prefer to leave his initials blank, we will refer to him as "your main partner".

What are the initials of or a nickname for your most recent male main partner?

____

What is your current agreement, if any, that you and [INITIALS/NICKNAME] have about having sex with partners outside of your relationship?

( ) Neither of us can have any sex with any outside partners

( ) We can have sex with outside partners, but with some conditions or restrictions that make it safer (for example, always using condoms OR refraining from certain acts OR knowing someone's HIV status)

( ) We can have sex with outside partners, without any conditions or restrictions

( ) We have no agreement

( ) I prefer not to answer

( ) Don't know

How long have you and [INITIALS/NICKNAME] had this agreement?

( ) Less than three months

( ) Three to six months

( ) 6 months – 1 year

( ) One to two years

( ) Two to three years

( ) Three to four years

( ) Four to five years

( ) More than 5 years

( ) I prefer not to answer

( ) Don't know

For each of the following, please indicate if this act IS allowed, IS NOT allowed, or if you do not have an agreement with [INITIALS/NICKNAME] about this act.

	**Allowed**	**Not allowed**	**No agreement**	**Don't know**
You topping someone else WITHOUT a condom	( )	( )	( )	( )
You bottoming someone else WITHOUT a condom	( )	( )	( )	( )
You topping someone else WITH a condom	( )	( )	( )	( )
You bottoming someone else WITH a condom	( )	( )	( )	( )

How many times in the past 12 months have you broken your current agreement with [INITIALS/NICKNAME]? Please enter "0" if this has not happened in the past 12 months.

____

During the times that you broke your agreement with [INITIALS/NICKNAME], which of the following did you do? Check all that apply.

[ ] Receptive anal sex (you bottomed with someone else)

[ ] Insertive anal sex (you topped with someone else)

[ ] Some other kind of sex

During the times that you had anal sex outside of your agreement, how often was it fully protected by a condom? This means that you or your partner used a condom throughout the entire sex act and the condom did not break or come off.

( ) Never

( ) Rarely

( ) Sometimes

( ) Often

( ) Always

( ) Prefer not to answer

( ) Don't know

To your knowledge, how many times in the past 12 months has [INITIALS/NICKNAME] broken your current agreement? Please enter "0" if this has not happened in the past 12 months.

____

Exchange Sex and Social Behavior

In the past 12 months, have you exchanged things like money or drugs for sex with a male partner? Check all that apply.

[ ] No

[ ] Yes, I gave a sex partner things like drugs or money for sex

[ ] Yes, a sex partner gave me things like drugs or money for sex

[ ] I prefer not to answer

[ ] Don't know

In the past 12 months, how often have you gone to a place (not online) where gay men hangout, meet or socialize? These could include bars, clubs, social organizations, parks, gay businesses, bookstores, sex clubs, etc.

( ) More than once a day

( ) Once a day

( ) More than once a week

( ) Once a week

( ) More than once a month

( ) Once a month

( ) Less than once a month

( ) Never

( ) I prefer not to answer

( ) Don't know

In the past 12 months, have you used any of following kinds of internet sites to meet or socialize with gay men?

[ ] Social network websites (such as Facebook)

[ ] Dating websites directed towards gay men

[ ] Mobile phone apps (such as gay chat, dating and hookup apps)

[ ] None of the above

[ ] I prefer not to answer

[ ] Don't know

In the past 12 months, how often did you use social network websites (such as Facebook) to meet or socialize with gay men?

( ) More than once a day

( ) Once a day

( ) More than once a week

( ) Once a week

( ) More than once a month

( ) Once a month

( ) Less than once a month

( ) Never

( ) I prefer not to answer

( ) Don't know

In the past 12 months, how often have you used dating websites directed towards gay men to meet or socialize with gay men?

( ) More than once a day

( ) Once a day

( ) More than once a week

( ) Once a week

( ) More than once a month

( ) Once a month

( ) Less than once a month

( ) Never

( ) I prefer not to answer

( ) Don't know

In the past 12 months, how often did you use mobile phone apps (such as gay chat, dating and hookup apps) to meet or socialize with gay men?

( ) More than once a day

( ) Once a day

( ) More than once a week

( ) Once a week

( ) More than once a month

( ) Once a month

( ) Less than once a month

( ) Never

( ) I prefer not to answer

( ) Don't know

How satisfied are you with your current sex life?

( ) Very satisfied

( ) Satisfied

( ) Unsure

( ) Dissatisfied

( ) Very Dissatisfied

( ) Don't know

( ) I prefer not to answer

Substance Use: Injection Drug Use

Have you ever in your life shot up or injected any drugs other than those prescribed for you? By shooting up, we mean anytime you might have used drugs with a needle, either by mainlining, skin popping, or muscling.

( ) No

( ) Yes

( ) I prefer not to answer

( ) Don't know

In the past 12 months, on average, how often did you inject?

( ) More than once a day

( ) Once a day

( ) More than once a week

( ) Once a week

( ) More than once a month

( ) Once a month

( ) Less than once a month

( ) Never

( ) I prefer not to answer

( ) Don't know

Which drug do you inject most often?

( ) Speedball - Heroin and cocaine together

( ) Heroin, by itself

( ) Cocaine, by itself

( ) Crack

( ) Crystal, meth, tina, crank, ice

( ) Something else (Specify)

( ) I prefer not to answer

( ) Don't know

Substance Use: Non-Injection Drug Use

The next questions are about drugs that you may have used but did not inject.

In the past 12 months, have you used any non-injection drugs (drugs you did not inject), other than those prescribed for you.

( ) No

( ) Yes

( ) I prefer not to answer

( ) Don't know

In the past 12 months, which drugs that were not prescribed to you did you use? (Check all that apply.)

[ ] Marijuana

[ ] Powdered cocaine (smoked or snorted)

[ ] Poppers (amyl nitrate)

[ ] X or Ecstasy

[ ] Painkillers (Oxycontin, Vicodin, Percocet)

[ ] Downers (Valium, Ativan, Xanax)

[ ] Crystal meth (tina, crank, ice)

[ ] Hallucinogens (LSD, mushrooms)

[ ] Special K (ketamine)

[ ] GHB

[ ] Crack cocaine

[ ] Other drug: ____

[ ] Heroin (smoked or snorted)

[ ] I prefer not to answer

[ ] Don't know

Drug use frequency

In the past 12 months, how often did you use [DRUGS USED]?

( ) More than once a day

( ) Once a day

( ) More than once a week

( ) Once a week

( ) More than once a month

( ) Once a month

( ) Less than once a month

( ) I prefer not to answer

( ) Don't know

In the past 12 months, have you been prescribed marijuana and had it filled at a legal dispensary?

( ) No

( ) Yes

( ) I prefer not to answer

( ) Don't now

Alcohol and Drugs at Last Sex

Before or during the last time you had sex with [INITIALS/NICKNAME], did you use:

( ) Alcohol

( ) Drugs

( ) Both alcohol and drugs

( ) Neither one

( ) I prefer not to answer

( ) Don't know

For the next set of questions, a drink of alcohol is a 12 oz beer, a 5 oz glass of wine, or a 1.5 ox shot of liquor. A 40 oz beer would count as 3 drinks. A cocktail with 2 shots would count as 2 drinks.

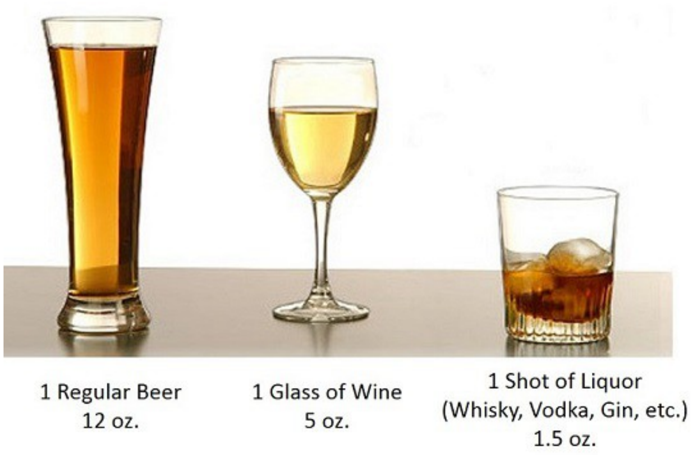

Before or during sex the last time you had sex with [INITIALS/NICKNAME], how many alcoholic drinks did you have?

____

The last time you had sex with [INITIALS/NICKNAME], which drugs did you use? Check all that apply.

[ ] *OPTIONS FROM PREVIOUS DRUG USE QUESTION*

[ ] Other drug: ____

[ ] I prefer not to answer

[ ] Don't know

HIV Testing

Have you ever been tested for HIV? An HIV test checks whether someone has the virus that causes AIDS.

( ) No

( ) Yes

( ) I prefer not to answer

( ) Don't know

In the past 2 years, that is since [system("date"),format="F"] of 2013, how many times have you been tested for HIV?

____

When did you have your most recent HIV test?

Month:

( ) January

( ) February

( ) March

( ) April

( ) May

( ) June

( ) July

( ) August

( ) September

( ) October

( ) November

( ) December

( ) Don't know

( ) Prefer not to answer

Year:: ____

Have you had an HIV test in the past 12 months [SURVEY DATE] of 2014)?

( ) Yes

( ) No

( ) Don't know

( ) I prefer not to answer

When you got tested in [*MONTH / YEAR OF HIV TEST*], where did you get tested?

( ) Private doctor's office

( ) HIV counseling and testing site

( ) Public health clinic/community health clinic

( ) Street outreach program/mobile unit

( ) Sexually transmitted disease clinic

( ) Hospital (inpatient)

( ) Correctional facility (jail or prison)

( ) Emergency room

( ) At home

( ) Other

( ) I prefer not to answer

( ) Don't know

HIV Status

What was the result of your most recent HIV test?

( ) Negative

( ) Positive

( ) Never obtained results

( ) Indeterminate

( ) I prefer not to answer

( ) Don't know

Before your test in [*MONTH / YEAR OF HIV TEST*], did you ever test positive for HIV?

( ) No

( ) Yes

( ) I prefer not to answer

( ) Don't know

Was your test in [*MONTH / YEAR OF HIV TEST*] your first positive test?

( ) No

( ) Yes

( ) I prefer not to answer

( ) Don't know

When did you first test positive?

Month

( ) January

( ) February

( ) March

( ) April

( ) May

( ) June

( ) July

( ) August

( ) September

( ) October

( ) November

( ) December

Year: ____

Are you currently taking antiretroviral medicines to treat your HIV infection?

( ) No

( ) Yes

( ) I prefer not to answer

( ) Don't know

What is the main reason you are not currently taking any antiretroviral medicines?

( ) Not currently going to a health care provider for my HIV

( ) CD4 count and viral load are good

( ) Don't have money or insurance for antiretroviral medicines

( ) Don't want to take antiretroviral medicines

( ) Other

( ) I prefer not to answer

( ) Don't know

At-home HIV Testing

Have you ever heard about home HIV tests?

( ) No

( ) Yes

( ) I prefer not to answer

Have you ever used a home HIV test?

( ) No

( ) Yes

( ) I prefer not to answer

In the past 12 months, how many times have you tested yourself with a home HIV test?

____

In the past 12 months, which of these home HIV tests have you used? Check all that apply.

[ ] Home Access HIV- Test System (where you prick your finger, collect a blood sample on a card, and mail that card to a lab for testing)

[ ] Oraquick In-Home HIV Test (where you collect your own oral fluid sample by swabbing your mouth, use the testing device yourself, and read the test results within 20 minutes)

[ ] Other rapid HIV test (please specify): ____

[ ] Don't know

[ ] I prefer not to answer

Under which circumstances did you use the home HIV test? Check all that apply.

[ ] I used it to test myself regularly

[ ] I used it to test myself before having sex with a new partner

[ ] I asked my sex partner to test himself/herself before having sex with me

[ ] I used it to test myself after having sex with someone I knew was HIV negative

[ ] I used it to test myself after having sex with someone I knew was HIV positive OR whose HIV status I did not know

[ ] I prefer not to answer

[ ] Don't know

[ ] Other: ____

You mentioned you used [*HOME HIV TEST*] to test yourself for HIV. Where did you get the home HIV test(s)? Check all that apply.

[ ] Bought Online

[ ] From a pharmacy

[ ] From a friend

[ ] From a sex partner

[ ] From my doctor’s office

[ ] From an HIV counseling and testing site

[ ] From a Health Department

[ ] From another research study

[ ] Other location or person (Specify (do not include someone’s name))

[ ] I prefer not to answer

[ ] Don't know

HIV Testing Intentions and Perceptions

How sure are you that you could get an HIV test within the next 3-6 months if you wanted to?

( ) Very sure I could not

( ) Somewhat sure I could not

( ) Slightly sure I could

( ) Somewhat sure I could

( ) Very sure I could

( ) I prefer not to answer

( ) Refuse to answer

How likely is it that you will get an HIV test within the next 3-6 months?

( ) Very unlikely

( ) Somewhat unlikely

( ) Slightly likely

( ) Somewhat likely

( ) Very likely

( ) I'd prefer not to answer

( ) Refuse to answer

Do you agree or disagree with the following statement:

Most gay men I know get tested for HIV at least every 3-6 months

( ) Strongly agree

( ) Agree

( ) Neither agree nor disagree

( ) Disagree

( ) Strongly disagree

( ) I prefer not to answer

( ) Don't know

PrEP and PEP

Pre-exposure prophylaxis (PrEP) is taking an antiretroviral pill, also called Truvada, every day for months or years to reduce a persons chance of getting HIV.

Before today, have you ever heard of people who do not have HIV taking PrEP, the antiretroviral medicine taken every day for months or years to reduce the risk of getting HIV?

( ) No

( ) Yes

Before today, have you ever heard of people who do <u>not</u> have HIV taking PrEP?

( ) No

( ) Yes

( ) Don't know

( ) I prefer not to answer

Before today, have you ever heard of PrEP?

( ) No

( ) Yes

( ) Don't know

( ) I prefer not to answer

In the past 12 months, have you had a discussion with a health care provider about taking PrEP?

( ) No

( ) Yes

( ) Don't know

( ) I prefer not to answer

In the past 12 months, when you discussed taking PrEP with a health care provider, did you receive the medicine or a prescription for the medicine?

( ) No

( ) Yes

( ) Don't know

( ) I prefer not to answer

In the past 12 months, have you taken PrEP to reduce the risk of getting HIV?

( ) No

( ) Yes

( ) Don't know

( ) Prefer not to answer

When you were on PrEP in the past 12 months, how frequently were you taking PrEP?

( ) Less than every day

( ) Every day

( ) Don't know

( ) Prefer not to answer

When you were on PrEP in the past 12 months, how many months in a row were you taking it?

( ) Less than 2 months

( ) 2 to 6 months

( ) 7 to 12 months

( ) Don't know

( ) Prefer not to answer

PrEP, continued

PrEP stands for pre-exposure prophylaxis. It involves a healthy person taking a pill used to treat HIV in order to prevent being infected with HIV. The pills have to be taken once a day, every day. Some people who take these pills experience side effects. These may include nausea and weight loss, which usually go away after the first month. In rare cases, taking the pill for long periods may damage the kidneys. The medication is prescribed by a doctor. Taking this medication provides only partial protection against HIV infection. So, a person on the medication should still practice other HIV prevention strategies like using condoms every time. For people who take the pill every day, studies have shown that it provides up to 90% protection against HIV infection.

Would you be willing to take anti-HIV medicines every day to lower your chances of getting HIV?

( ) No

( ) Yes

( ) I prefer not to answer

( ) Don't know

PEP

In the past 12 months, have you taken PEP to reduce the risk of getting HIV?

( ) No

( ) Yes

( ) Don't know

( ) I prefer not to answer

Conversations with partners - HIV Positive respondents

In the last 6 months, how many times have you talked with [INITIALS/NICKNAME] about:

	**Never**	**1-2 times**	**3-4 times**	**5 or more times**
Your antiviral medication adherence	( )	( )	( )	( )
Your viral load or viral suppression	( )	( )	( )	( )
Scheduling your HIV provider visits	( )	( )	( )	( )
Attending your HIV provider visits	( )	( )	( )	( )
PrEP to protect [INITIALS/NICKNAME]? Pre-exposure prophylaxis (PrEP) is taking an antiretroviral pill, also called Truvada, every day for months or years to reduce a persons chance of getting HIV.	( )	( )	( )	( )

In the last 6 months, how many times have you talked with [INITIALS/NICKNAME] about:

	**Never**	**1-2 times**	**3-4 times**	**5 or more times**
PrEP to protect [INITIALS/NICKNAME]? Pre-exposure prophylaxis (PrEP) is taking an antiretroviral pill, also called Truvada, every day for months or years to reduce a persons chance of getting HIV.	( )	( )	( )	( )
Your antiviral adherence to protect [INITIALS/NICKNAME]?	( )	( )	( )	( )
Consistent condom use to protect [INITIALS/NICKNAME]?	( )	( )	( )	( )
HIV testing or test results for [INITIALS/NICKNAME]?	( )	( )	( )	( )
[INITIALS/NICKNAME] refraining from receptive anal sex (bottoming)?	( )	( )	( )	( )

Conversations with partners - HIV negative respondents

In the last 6 months, how many times have you talked with [INITIALS/NICKNAME] about:

	Never	1-2 times	3-4 times	5 or more times
[INITIALS/NICKNAME]'s antiviral medication adherence	( )	( )	( )	( )
[INITIALS/NICKNAME]'s viral load or viral suppression	( )	( )	( )	( )
Scheduling [INITIALS/NICKNAME]'s HIV provider visits	( )	( )	( )	( )
Attending [INITIALS/NICKNAME]'s HIV provider visits	( )	( )	( )	( )
PrEP to protect you?	( )	( )	( )	( )
Consistent condom use to protect you?	( )	( )	( )	( )
HIV testing or your test results?	( )	( )	( )	( )
You refraining from receptive anal sex (bottoming)?	( )	( )	( )	( )

Acute Infection

Do you think that after a person is infected with HIV, they can show symptoms within a month of being infected?

( ) No

( ) Yes

( ) Don't know

Experiences of Stigma

During the past 12 months, have any of the following things happened to you because someone knew or assumes you were attracted to men?

	No	Yes	I prefer not to answer	Don't Know
You were called names or insulted	( )	( )	( )	( )
You received poorer services than other people in restaurants, stores, other businesses or agencies	( )	( )	( )	( )
You were treated unfairly at work or school	( )	( )	( )	( )

You were denied or given lower quality health care	( )	( )	( )	( )
You were physically attacked or injured	( )	( )	( )	( )

How strongly do you agree or disagree with the following statement: "Most people in my area are tolerant of gays and bisexuals."

( ) Strongly agree

( ) Agree

( ) Neither agree nor disagree

( ) Disagree

( ) Strongly disagree

( ) I prefer not to answer

( ) Don't know

How strongly do you agree or disagree with each statement below?

Most people in my area would discriminate against someone with HIV.

( ) Strongly agree

( ) Agree

( ) Neither agree nor disagree

( ) Disagree

( ) Strongly disagree

( ) I prefer not to answer

( ) Don't know

Most people in my area would support the rights of a person with HIV to live and work wherever they wanted to.

( ) Strongly agree

( ) Agree

( ) Neither agree nor disagree

( ) Disagree

( ) Strongly disagree

( ) I prefer not to answer

( ) Don't know

Most people in my area would not be friends with someone with HIV.

( ) Strongly agree

( ) Agree

( ) Neither agree nor disagree

( ) Disagree

( ) Strongly disagree

( ) I prefer not to answer

( ) Don't know

Most people in my area would think that people who got HIV through sex or drug use have gotten what they deserve.

( ) Strongly agree

( ) Agree

( ) Neither agree nor disagree

( ) Disagree

( ) Strongly disagree

( ) I prefer not to answer

( ) Don't know

Stigma module 2

The next questions are about things you may have ever experienced because someone knew or assumed you have sex with men.

	Yes	No	Doesn't Apply	I prefer not to answer	Don't know
Have you ever felt excluded from family gatherings because you have sex with men?	( )	( )	( )	( )	( )
Have you ever felt that family members have made discriminatory remarks or gossiped about you because you have sex with men?	( )	( )	( )	( )	( )
Have you ever felt rejected by your friends because you have sex with men?	( )	( )	( )	( )	( )
Have you ever felt afraid to go to health care services because you worry someone may learn you have sex with men?	( )	( )	( )	( )	( )
Have you ever felt that health care providers did not treat you well or gave	( )	( )	( )	( )	( )

you lower quality care because you have sex with men?					
Have you ever avoided going to health care services because you worried someone may learn you have sex with men?	( )	( )	( )	( )	( )
Have you ever heard health care providers make discriminatory remarks or gossip about you because you have sex with men?	( )	( )	( )	( )	( )
Have you ever felt that the police refused to protect you because you have sex with men?	( )	( )	( )	( )	( )

The next questions are about things you may have ever experienced because someone knew or assumed you have sex with men.

	**Yes**	**No**	**I prefer not to answer**	**Don't know**
Have you ever felt scared to walk around in public places because you have sex with men?	( )	( )	( )	( )
Have you ever been verbally harassed because you have sex with men?	( )	( )	( )	( )
Have you ever been blackmailed by someone because you have sex with men?	( )	( )	( )	( )
Have you ever been physically aggressed (pushed, shoved, slapped, hit, kicked, choked or physically hurt) because you have sex with men?	( )	( )	( )	( )
Have you ever been forced to have sex when you did not want to? By forced, I mean physically forced, coerced to have sex, or penetrated with an object when you did not want to.	( )	( )	( )	( )

Do you feel you were forced to have sex because you have sex with men?

( ) Yes

( ) No

( ) I prefer not to answer

( ) Don't know

Adverse Childhood Events

How often did a parent, stepparent, or adult living in your home...

	Never	Once or twice	Sometimes	Often	Very often	I prefer not answer	Don't know
Swear at you, insult you, or put you down?	( )	( )	( )	( )	( )	( )	( )
Threaten to hit you or throw something at you, but didn't do it?	( )	( )	( )	( )	( )	( )	( )

Sometimes parents or other adults hurt children.
While you were growing up, that is, in your first 18 years of life, how often did a parent, stepparent or adult living in your home:

	Never	Once or twice	Sometimes	Often	Very often	I prefer not answer	Don't know
Push, grab, slap, or throw something at you?	( )	( )	( )	( )	( )	( )	( )
Hit you so hard that you had marks or were injured?	( )	( )	( )	( )	( )	( )	( )

Some people, while they are growing up in their first 18 years of life, had a sexual experience with an adult or someone at least 5 years older than themselves. These experiences may have involved a relative, family friend, or stranger.

During the first 18 years of life, did an adult, relative, family friend, or stranger ever:

Touch or fondle your body in a sexual way?

( ) No

( ) Yes

( ) I prefer not answer

( ) Don't know

Have you touch their body in a sexual way?

( ) No

( ) Yes

( ) I prefer not answer

( ) Don't know

Attempt to have any type of sexual intercourse (oral or anal) with you?

( ) No

( ) Yes

( ) I prefer not answer

( ) Don't know

Actually have any type of sexual intercourse (oral or anal) with you?

( ) No

( ) Yes

( ) I prefer not answer

( ) Don't know

While you were growing up in your first 18 years of life, how often did your father (or stepfather) or mother's boyfriend do any of these things to you mother (or stepmother):

	**Never**	**Once or twice**	**Sometime s**	**Often**	**Very often**	**I prefer not to answer**	**Don't know**
Push, grab, slap or throw something at her?	( )	( )	( )	( )	( )	( )	( )

Kick, bite, hit her with a fist, or hit her something hard?	( )	( )	( )	( )	( )	( )	( )
Repeatedly hit her over at least a few minutes?	( )	( )	( )	( )	( )	( )	( )
Threaten her with a knife or gun, or use a knife to hurt her?	( )	( )	( )	( )	( )	( )	( )

Did you live with anyone who was a problem drinker or alcoholic?

( ) No

( ) Yes

( ) I prefer not answer

( ) Don't know

Did you live with anyone who used street drugs?

( ) No

( ) Yes

( ) I prefer not answer

( ) Don't know

Was a household member depressed or mentally ill?

( ) No

( ) Yes

( ) I prefer not answer

( ) Don't know

Did a household member attempt suicide?

( ) No

( ) Yes

( ) I prefer not answer

( ) Don't know

Did a household member go to prison?

( ) No

( ) Yes

( ) I prefer not to answer

( ) Don't know

While you were growing up, in your first 18 years of life, were your parents ever separated or divorced?

( ) No

( ) Yes

( ) I prefer not to answer

( ) Don't know

STIs

Viral STI Diagnoses

Has a doctor, nurse or other health care provider ever told you that you had any of the following? Check all that apply.

[ ] Hepatitis

[ ] Genital herpes

[ ] Genital warts

[ ] Human papillomavirus or HPV

[ ] None of the above

[ ] I prefer not to answer

[ ] Don't know

What type or types of hepatitis have you had? Check all that apply.

[ ] Hepatitis A

[ ] Hepatitis B

[ ] Hepatitis C

[ ] Other

[ ] I prefer not to answer

[ ] Don't know

There are vaccines or shots that can prevent some types of hepatitis. Have you ever had a hepatitis vaccine?

( ) No

( ) Yes

( ) I prefer not to answer

( ) Don't know

What type or types of hepatitis vaccine have you had?

( ) Hepatitis A vaccine

( ) Hepatitis B vaccine

( ) Both Hepatitis A and B vaccine

( ) I prefer not to answer

( ) Don't know

Bacterial STI Diagnoses

In the past 12 months [SURVEY DATE] of 2014), has a doctor, nurse or other health care provider told you that you had any of the following? Check all that apply.

[ ] Gonorrhea

[ ] Chlamydia

[ ] Syphilis

[ ] None of the above

[ ] I prefer not to answer

[ ] Don't know

Bacterial STI Testing

Even though a doctor, nurse, or other health care provider did not tell you that you had gonorrhea, in the past 12 months [SURVEY DATE] of 2014) were you tested for gonorrhea?

( ) No

( ) Yes

( ) I prefer not to answer

( ) Don't know

Even though a doctor, nurse, or other health care provider did not tell you that you had chlamydia, in the past 12 months [SURVEY DATE] of 2014) were you tested for chlamydia?

( ) No

( ) Yes

( ) I prefer not to answer

( ) Don't know

Even though a doctor, nurse, or other health care provider did not tell you that you had syphilis, in the past 12 months [SURVEY DATE] of 2014) were you tested for syphilis?

( ) No

( ) Yes

( ) I prefer not to answer

( ) Don't know

HPV

A vaccine to prevent human papillomavirus (HPV) infection is available and is called the HPV shot, cervical cancer vaccine, GARDASIL®, or CERVARIX®. Have you ever received the HPV vaccine?

( ) No

( ) Yes

( ) I prefer not to answer

( ) Don't know

How old were you when you received your first dose of the HPV vaccine?

Prevention Activities

In the past 12 months, have you gotten any free condoms, not counting those given to you by a friend, relative, or sex partner?

( ) No

( ) Yes

( ) I prefer not to answer

( ) Don't know

In the past 12 months, have you had a one-on-one conversation with an outreach worker, counselor, or prevention program worker about ways to prevent HIV? Don't count the times where you had a conversation as part of an HIV test.

( ) No

( ) Yes

( ) I prefer not to answer

( ) Don't know

In the past 12 months, have you been a participant in any organized session(s) involving a small group of people to discuss ways to prevent HIV? Don't include discussions you had with a group of friends.

( ) No

( ) Yes

( ) I prefer not to know

( ) Don't know

Condom Fit Questions - Randomization 1

How much protection do you think you get from HIV with using a condom for anal sex, versus not using a condom for anal sex?

0 ________________________[__]_____________________________ 100

At what level of protection against HIV would you NOT be willing to use condoms?

0 ________________________[__]_____________________________ 100

Condom Fit Questions - Randomization 2

How often do you think condoms fail (slip or break) when used for anal sex?

0 ________________________[__]_____________________________ 100

At what amount of condom failure (slip or break) would you NOT be willing to use condoms for anal sex?

0 ________________________[__]_____________________________ 100

Condom Fit Questions for all

Currently, there is no condom that is approved by the FDA for use during anal sex. If a condom was FDA-approved for anal sex, would you be more likely to use condoms every time you have anal sex?

( ) Yes

( ) No

If a condom was labeled by the FDA as "more pleasurable," would you be more likely to use this condom for anal sex?

( ) Yes

( ) No

Prevention Campaigns

Let's stop HIV together:

( ) Never

( ) Rarely

( ) Sometimes

( ) Often

( ) Very Often

( ) I prefer not to answer

( ) Don't know

On a scale of 0 to 5, where 0 means "not very effective" and 5 means "very effective", how effective do you think this slogan or message is?

( ) 0 (Not Effective)

( ) 1

( ) 2

( ) 3

( ) 4

( ) 5 (Very Effective)

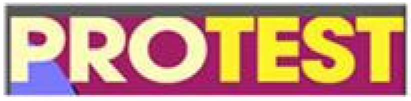

Protest:

( ) Never

( ) Rarely

( ) Sometimes

( ) Often

( ) Very Often

( ) I prefer not to answer

( ) Don't know

On a scale of 0 to 5, where 0 means "not very effective" and 5 means "very effective", how effective do you think this slogan or message is?

( ) 0 (Not Effective)

( ) 1

( ) 2

( ) 3

( ) 4

( ) 5 (Very Effective)

ACT Against AIDS:

( ) Never

( ) Rarely

( ) Sometimes

( ) Often

( ) Very Often

( ) I prefer not to answer

( ) Don't know

On a scale of 0 to 5, where 0 means "not very effective" and 5 means "very effective", how effective do you think this slogan or message is?

( ) 0 (Not Effective)

( ) 1

( ) 2

( ) 3

( ) 4

( ) 5 (Very Effective)

Greater than AIDS:

( ) Never

( ) Rarely

( ) Sometimes

( ) Often

( ) Very Often

( ) I prefer not to answer

( ) Don't know

On a scale of 0 to 5, where 0 means "not very effective" and 5 means "very effective", how effective do you think this slogan or message is?

( ) 0 (Not Effective)

( ) 1

( ) 2

( ) 3

( ) 4

( ) 5 (Very Effective)

Start Talking. Stop HIV.

( ) Never

( ) Rarely

( ) Sometimes

( ) Often

( ) Very Often

( ) I prefer not to answer

( ) Don't know

On a scale of 0 to 5, where 0 means "not very effective" and 5 means "very effective", how effective do you think this slogan or message is?

( ) 0 (Not Effective)

( ) 1

( ) 2

( ) 3

( ) 4

( ) 5 (Very Effective)

Testing Makes us Stronger:

( ) Never

( ) Rarely

( ) Sometimes

( ) Often

( ) Very Often

( ) I prefer not to answer

( ) Don't know

On a scale of 0 to 5, where 0 means "not very effective" and 5 means "very effective", how effective do you think this slogan or message is?

( ) 0 (Not Effective)

( ) 1

( ) 2

( ) 3

( ) 4

( ) 5 (Very Effective)

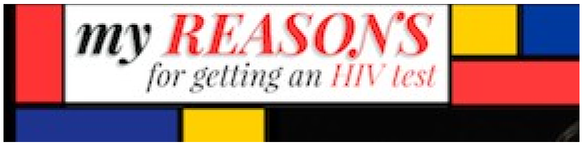

Reasons:

( ) Never

( ) Rarely

( ) Sometimes

( ) Often

( ) Very Often

( ) I prefer not to answer

( ) Don't know

On a scale of 0 to 5, where 0 means "not very effective" and 5 means "very effective", how effective do you think this slogan or message is?

( ) 0 (Not Effective)

( ) 1

( ) 2

( ) 3

( ) 4

( ) 5 (Very Effective)

HIV
TREATMENT
WORKS

HIV Treatment Works:

( ) Never

( ) Rarely

( ) Sometimes

( ) Often

( ) Very Often

( ) I prefer not to answer

( ) Don't know

On a scale of 0 to 5, where 0 means "not very effective" and 5 means "very effective", how effective do you think this slogan or message is?

( ) 0 (Not Effective)

( ) 1

( ) 2

( ) 3

( ) 4

( ) 5 (Very Effective)

As far as you know, did you participate in the "Sex is the Question" Survey between October 2014 and April 2015?

( ) Yes

( ) No

( ) I'm not sure

For this national study, we are recruiting a large number of men like you. Can you tell us the name of social networking website where we could reach other men like you who might like to complete this survey?

____

Future Contact

The PRISM Health team conducts many research projects at Emory University. Would you like to be contacted for potential participation in our future projects?

( ) Yes

( ) No

Please provide the email address you would like for us to use to contact you for future studies.

____

Survey End

Thank you for taking our survey! Your response is very important to us!

If you have any questions or comments, you may contact study staff at sexisthequestion@emory.edu.

To get more information about HIV, please visit: www.cdc.gov/hiv

Otherwise, you can close your browser.
