# Supplementary material for: The Annual American Men’s Internet Survey of Behaviors of Men Who Have Sex With Men in the United States: 2015 Key Indicators Report
Source: JMIR Public Health Surveill. 2017 Mar 25;3(1):e13. doi: 10.2196/publichealth.7119 (PMC5390111; doi:10.2196/publichealth.7119)
Supplement: Multimedia Appendix 2 [file publichealth_v3i1e13_app2.pptx]

## Slide 1
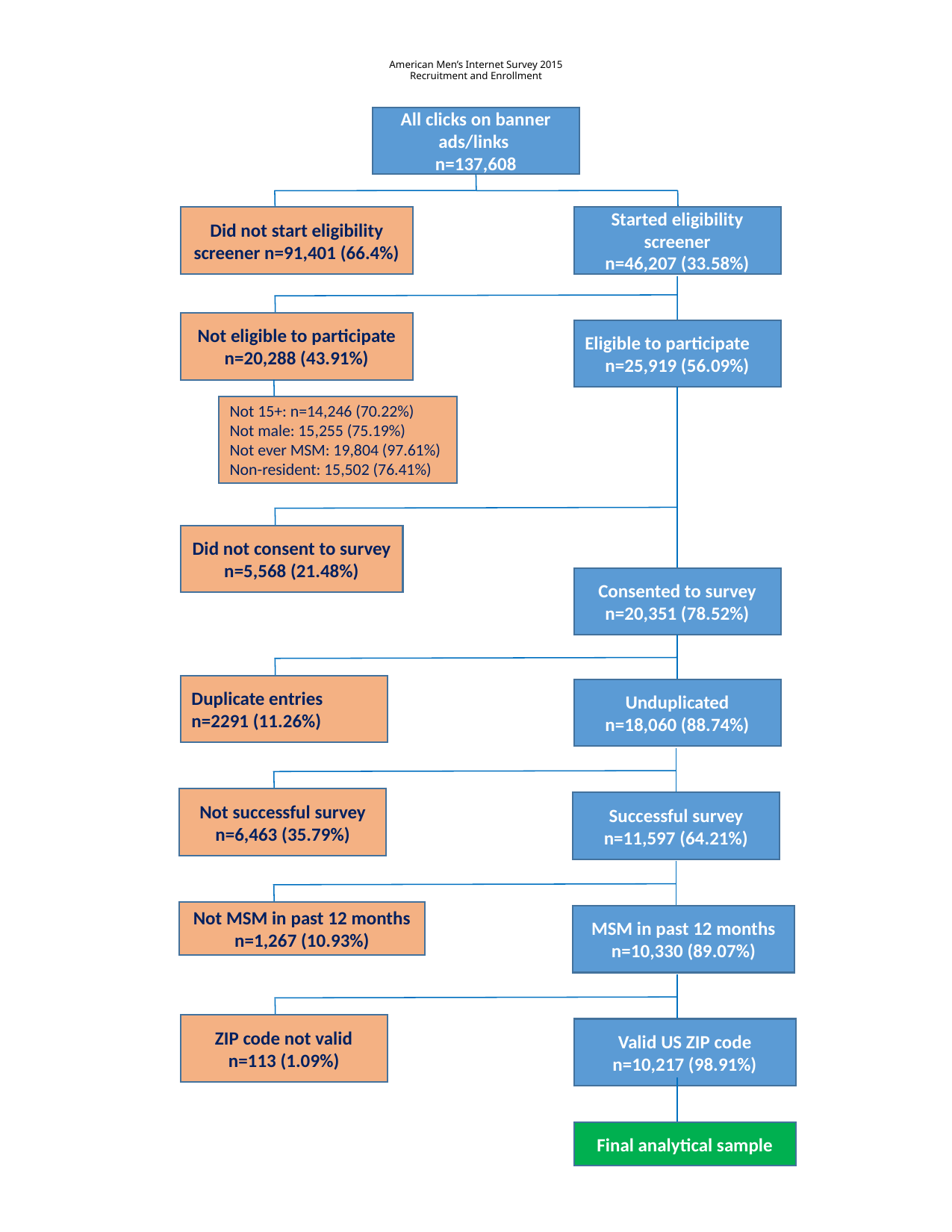

# American Men’s Internet Survey 2015 Recruitment and Enrollment
All clicks on banner ads/links
n=137,608
Did not start eligibility screener n=91,401 (66.4%)
Started eligibility screener
n=46,207 (33.58%)
Not eligible to participate
n=20,288 (43.91%)
Eligible to participate
n=25,919 (56.09%)
Not 15+: n=14,246 (70.22%)
Not male: 15,255 (75.19%)
Not ever MSM: 19,804 (97.61%)
Non-resident: 15,502 (76.41%)
Did not consent to survey
n=5,568 (21.48%)
Consented to survey
n=20,351 (78.52%)
Duplicate entries
n=2291 (11.26%)
Unduplicated
n=18,060 (88.74%)
Not successful survey
n=6,463 (35.79%)
Successful survey
n=11,597 (64.21%)
Not MSM in past 12 months
n=1,267 (10.93%)
MSM in past 12 months
n=10,330 (89.07%)
ZIP code not valid
n=113 (1.09%)
Valid US ZIP code
n=10,217 (98.91%)
Final analytical sample
